# Bacteriological Quality of Abattoir Effluents Discharged into Water Bodies in Abuja, Nigeria

**DOI:** 10.5402/2012/515689

**Published:** 2012-07-19

**Authors:** W. D. Nafarnda, I. E. Ajayi, J. C. Shawulu, M. S. Kawe, G. K. Omeiza, N. A. Sani, O. Z. Tenuche, D. D. Dantong, S. Z. Tags

**Affiliations:** ^1^Department of Veterinary Public Health and Preventive Medicine, Faculty of Veterinary Medicine, University of Abuja, PMB 117, Abuja 901001, Nigeria; ^2^Department of Veterinary Anatomy, Faculty of Veterinary Medicine, University of Abuja, PMB 117, Abuja 901001, Nigeria; ^3^Department of Veterinary Parasitology and Entomology, Faculty of Veterinary Medicine, University of Abuja, PMB 117, Abuja 901001, Nigeria; ^4^Department of Veterinary Pathology, Faculty of Veterinary Medicine, University of Abuja, PMB 117, Abuja 901001, Nigeria

## Abstract

Bacteriological characteristics of abattoir effluents (wastewater), abattoir water source, and water bodies receiving abattoir wastewater were investigated in Abuja, Nigeria using the multiple-tube fermentation technique. Source of water to the abattoirs and the usage of water bodies receiving abattoir effluents were determined using questionnaires. Bacterial counts ranged from 4.8 × 10^6^ to 5.8 × 10^5^ /100 mL of total coliform (TC), 8.2 × 10^4^ to 3.2 × 10^4^/100 mL of Fecal coliform (FC), 5.2 × 10^4^ to 2.0 × 10^4^/100 mL of *Fecal streptococcus *and 1.2 × 10^4^ to 2.0 × 10^3^/100 mL of *Escherichia coli* for abattoir effluents 6.6 × 10^5^ to 6.0 × 10^5^/100 mL of TC, 6.2 × 10^4^ to 1.8 × 10^4^/100 mL of FC, 1.8 × 10^4^ to 6.0 × 10^3^/100 mL of *F. streptococcus*, and 4.8 × 10^3^ to 6.6 × 10^2^/100 mL of *E. coli* for water bodies receiving abattoir effluents 100 m downstream. TC bacteria counts for abattoir effluents exceeded recommended limit for discharge into surface water in Nigeria. No significant difference (*P* < 0.05) was observed between bacterial counts of abattoir effluents and receiving water bodies 100 m downstream: an indication of contamination of receiving water bodies by abattoir effluents and possible public and environmental health hazards.

## 1. Introduction

In Nigeria, the abattoir industry is an important component of the livestock industry providing domestic meat supply to over 150 million people and employment opportunities for teaming population. However, the abattoir industries are less developed in developing countries like Nigeria. Facilities for the treatment of abattoir effluents are lacking, unlike in developed countries where these facilities are adequately provided [1]. Potential health risks from waterborne pathogens can exist in water contaminated by abattoir effluents [2], runoff from feedlots [3], dairy farms [4], grazed pastures [5, 6], fallow and sod amended with poultry litter [7], grassland treated with dairy manure [8], and sewage sludge treated land [9]. Such contamination of water bodies from abattoir wastes could constitute significant environmental and public health hazards [10–13].

Bacteria from abattoir waste discharged into water columns can subsequently be absorb to sediments, and when the bottom stream is disturbed, the sediment releases the bacteria back into the water columns presenting long-term health hazards [14]. Pathogens present in animal carcasses or shed in animal wastes may include rotaviruses, hepatitis E virus, *Salmonella *spp., *E. coli *O157 : H7, *Yersinia enterocolitica*, *Campylobacter *spp., *Cryptosporidium parvum*, and *Giardia lamblia* [15]. The primary reservoir for *E. coli *O157 : H7 has been reported to be healthy cattle in a study in Canada, although this bacterium is also endemic to swine and sheep [16]. These zoonotic pathogen can exceed millions to billions per gram of feces, and may infect humans through various routes such as contaminated air, contact with livestock animals or their waste products, swimming in water impacted by animal feces, exposure to potential vectors (such as flies, mosquitoes, water fowl, and rodents), or consumption of food or water contaminated by animal wastes [17, 18]. The consequences of infection by pathogens originating from animal wastes can range from temporary morbidity to mortality, especially in high-risk individuals. Due to the difficulties in quantifying pathogens, indicators of fecal pollution, including coliform bacteria, fecal coliforms, *E. coli*, and/or Enterococci have been monitored in lieu of overt pathogens for more than 100 years [19]. Epidemiological evidence supports the relationship between the fecal indicator bacteria *E. coli*, Enterococci, and the incidence of gastrointestinal illness following recreational water exposure and provides the basis for water quality regulations [20].

In Nigeria, abattoir wastes are sources of embarrassment that requires immediate remedy [21]. Abattoir wastes with large quantities of animal faeces are often channeled directly into water bodies, used for domestic purposes by human beings. In Port Harcourt area of Nigeria, abattoir effluents are channeled directly without treatment into one of the tributaries of the river Niger [22].

The bacteriological characteristics of abattoir wastewater and its possible effect on receiving water bodies which is likely to cause pollution with intensified environmental and public health hazards has not been documented in Abuja, Nigeria. The objective of this study was therefore, to investigate the bacteriological characteristics of abattoir wastewater, to determine its strength, its relationship with receiving water bodies and possible public/environmental health hazards in Abuja, Nigeria. The results are based on mean concentrations of total colifrom (TC), fecal coliform (FC), *Fecal streptococcus, and Escherichia coli* in samples of abattoir wastewater, water servicing the abattoirs, and receiving water bodies collected at 5 major abattoirs in Abuja. Data on the usage of receiving water bodies and sources of water servicing the abattoirs were also obtained. Results obtained could be helpful in defining future abattoir wastewater treatment and management practices in Nigeria and elsewhere.

## 2. Methods and Materials

Samples and data were collected from five major abattoirs in Abuja and water bodies receiving abattoir wastewater. Abuja is the federal capital of Nigeria with a population of about 1.857 million people and one of the fastest growing cities in the world [23]. It is located in the north central part of Nigeria, with coordinates of 9°4′0′′N 7°29′0′′E and a total land area of 713 km^2^(275.3 sq m). The 5 major abattoirs serving the city and its environs are the Deidei, Gwagwalada, Karu, Kubwa, and Kuje abattoirs. Animals slaughtered at the 5 major abattoirs include cattle, sheep, and goats, with average daily slaughter figures of 350 cattle, 450 sheep, and 670 goats. 

### 2.1. Sample Collection and Analyses

Samples were collected from Deidei, Gwagwalada, Karu, Kubwa, and Kuje abattoirs between the months of January and August 2011. The abattoirs were visited at 4 days interval (every market day of the area) to collect samples. A total of 60 samples were collected from each of the abattoirs during the period. Samples were collected in the morning during the peak activities between 08.00 am and 09.00 am using the grab sampling method with a wide mouthed 500 mL sterilized Pyrex glass bottles with tight screw dust proof stoppers. Wastewater samples were collected at the abattoirs from a point where it is thoroughly mixed and close to the discharging point (outlet) 100 mm below the surface, samples from receiving water bodies were collected 100 m upstream and downstream (before and after mixing with the abattoir wastewater), respectively. The distance was determined using a meter measuring tape. The bottles were filled leaving a top space of about 2.5 cm. Samples were stored on ice for transportation to the laboratory and between separation procedures. Samples were processed and incubated within 5 hours of sampling. Extracts from these samples were first diluted in peptone water 0*·*1%; 10 mL of the sample were added to 90 mL of the diluents, producing a dilution of 10^−1^. Successive decimal dilutions were obtained, and then prepared for the analyses of TC, FC, *E. coli, and F. streptococcus* using the multiple-tube most probable number (MPN) fermentation technique. The presumptive, confirmed and completed tests were carried out as described in the Standard Methods for the Examination of Water and Wastewater, American Public Health Association [24]. The numbers of positive findings were enumerated and statistical tables (MPN tables) were used to determine bacteria counts. 

A well structured questionnaire was orally administered to obtain data on the water servicing the abattoirs and the usage of water bodies receiving abattoir wastewater. The distance from abattoir wastewater outlet to water bodies and the point of usage of the receiving water bodies were measured using a meter measuring tape at each abattoir. Information on abattoir wastewater treatment methods before discharging was obtained using a questionnaire.

The mean value concentrations of TC, FC, *F. streptococcus*, and* E. coli* (MPN/100 mL) for abattoir wastewater, water servicing the abattoirs, and water bodies receiving abattoir wastewater were determined. Microbial concentrations of the abattoir wastewater, water servicing the abattoirs, upstream and downstream water bodies receiving abattoir wastewater were compared (*P* < 0.05) to establish if there is any relationship. The TC counts of the abattoir wastewater were also compared with the Federal Environmental Protection Agency (FEPA) [25] recommended level for the discharge of industrial wastewater into surface water and land application in Nigeria.

## 3. Results

Water servicing the abattoirs were from borehole, well and stream water at Deidei, Karu, and Kuje abattoirs while borehole and well water were used at Gwagwalada and Kubwa abattoirs. Untreated abattoir wastewater was discharged directly into water bodies (streams, rivers, and drainages) that are used for drinking, bathing, washing of clothes, home utensils, watering animals, watering of crops, and other domestic purposes downstream at distances of 680 m at Deidei, 415 m at Gwagwlada, 610 m at Karu, 542 m at kubwa, and 320 m at kuje area. 

Tables 1 to 5 present the mean values of TC, FC, *F. streptococcus* and *E. coli* (MPN/100 mL) of the abattoir wastewater, water servicing the abattoirs, water bodies receiving abattoir wastewater at 100 m upstream and downstream and the recommended TC count of industrial effluents (wastewater) limit for discharge into surface water and land application in Nigeria. 

## 4. Discussion

This study was conducted to determine the bacteriological characteristics of abattoir effluents discharged into water bodies and its possible health hazards on receiving water bodies in Abuja, Nigeria. The concentrations of TC, FC, *F. streptococcus*, and *E. coli *were determined in the abattoir effluents, water servicing the abattoir, and water bodies receiving abattoir effluents. Bacterial concentrations (MPN/100 mL) of abattoir effluents ranged from 4.8 × 10^6^ to 5.8 × 10^5^/100 mL of TC, 8.2 × 10^4^ to 3.2 × 10^4^/100 mL of FC, 5.2 × 10^4^ to 2.0 × 10^4^/100 mL of *F. streptococcus*, and 1.2 × 10^4^ to 2.0 × 10^3^/100 mL of *E. coli* while receiving water bodies ranged from 6.2 × 10^3^ to 1.4 × 10^3^/100 mL of TC, 2.6 × 10^3^ to 2.2 × 10^2^/100 mL of FC, 1.2 × 10^3^ to 1.2 × 10^2^/100 mL of *F. streptococcus*, and 2.0 × 10^2^ to 4.0 × 10^1^/100 mL of *E. coli* 100 m upstream, and 6.6 × 10^5^ to 6.0 × 10^5^/100 mL of TC, 6.2 × 10^4^ to 1.8 × 10^4^/100 mL of FC, 1.8 × 10^4^ to 6.0 × 10^3^/100 mL of *F. streptococcus*, and 4.8 × 10^3^ to 6.6 × 10^2^/100 mL of *E. coli* 100 m downstream after mixing with the abattoir wastewater. Bacterial counts of 4.8 × 10^2^ to 1.2 × 10^2^/100 mL of TC, 4.0 × 10^2^ to 2.6 × 10/100 mL of FC, 1.8 × 10^2^ to 2.1 × 10/100 mL of *F. streptococcus*, and 4.8 × 10 to 1.2 × 10/100 mL of *E. coli* were observed in the water sources servicing the abattoir (Tables 1–5). TC bacteria concentration in the abattoir wastewater discharged exceeded the recommended limit for the discharge of effluents into water bodies and land application in Nigeria [25]. There was no significant difference (*P* < 0.05) between the mean bacterial counts of abattoir wastewater and receiving water bodies 100 m downstream; is an indication of contamination of receiving water bodies with abattoir wastewater, similar findings has been reported in other places [2, 21, 26, 27]. The receiving water bodies were used for drinking, bathing, washing, watering of animals, watering of crops, and other domestic purpose downstream. 

Fecal coliforms live in the digestive tract of warm-blooded animals; their counts are often used as a surrogate measurement for gastro-enteric pathogens, since the presence of fecal coliform bacteria is an indication of contamination by human and/or animal wastes. *E. coli* is the most prevalent member of the fecal coliform group; livestock harbour the bacteria and release it in their feces. And so the presence of *E. coli* in water is considered a specific indicator of fecal contamination and the presence of enteric pathogens;it is used as the general indicator organism that signals whether there has been fecal contamination or not. The high levels of total coliforms and *E. coli* in the abattoir wastewater and receiving water bodies are therefore an indication of the contamination of water sources with feacal material and possibly pathogenic organisms from abattoir wastewater discharged untreated; similar findings have early been reported [2–7]. The discharge of untreated abattoir wastewater could result in out breaks of *E. coli *infection as observed by [28–30]. Bacterial pathogens such as Salmonella [31], Campylobacter, and *Listeria monocytogenes* [32] have been isolated from abattoir wastewater. The microbial concentrations observed upstream in this study could be as a result of indiscriminate disposal of domestic wastes into water bodies by human beings in these areas. 

## 5. Conclusion

This study observed that untreated abattoir wastewater discharged into water bodies in Abuja, Nigeria contains bacterial counts above the recommended level for discharge into water bodies in Nigeria. Receiving water bodies were contaminated with bacteria pathogens that could impact on public health, especially that streams and rivers still serves as major sources of water supply in developing countries like Nigeria. The importance of adopting appropriate abattoir wastewater treatment measures to prevent the chances of contaminating water bodies and ground water in Nigeria is therefore recommended. Determination of specific pathogenic microorganisms in abattoir wastewater and their health impacts is recommended.

## Figures and Tables

**Table 1:** Bacteriological characteristics of Deidei abattoir wastewater, source of water used at the abattoir, receiving water bodies upstream and downstream, TC effluents limit for discharge into water bodies and land application in Nigeria (MPN/100 mL).

Parameter	Mean value of abattoir wastewater	Mean value of water used at the abattoir	Mean value of water body upstream	Mean value of water body downstream	^ ∗^Effluent limit for discharge into surface water in Nigeria	^ ∗^Effluent limit for discharge into land application in Nigeria
TC	3.2 × 10^6^	2.2 × 10^3^	6.2 × 10^3^	6.6 × 10^5^	4.0 × 10^2^	5.0 × 10^2^
FC	6.2 × 10^4^	2.8 × 10^2^	2.6 × 10^3^	3.4 × 10^4^	—	—
*F. streptococcus*	2.1 × 10^4^	4.5 × 10	1.2 × 10^2^	1.0 × 10^4^	—	—
*E. coli*	3.2 × 10^3^	2.8 × 10	1.0 × 10^2^	2.1 × 10^3^	—	—

**Table 2:** Bacteriological characteristics of Gwagwalada abattoir wastewater, source of water used at the abattoir, receiving water bodies upstream and downstream, TC effluents limit for discharge into water bodies and land application in Nigeria (MPN/100 mL).

Parameter	Mean value of abattoir wastewater	Mean value of water used at the abattoir	Mean value of water body upstream	Mean value of water body downstream	^ ∗^Effluent limit for discharge into surface water in Nigeria	^ ∗^Effluent limit for discharge into land application in Nigeria
TC	8.2 × 10^5^	1.2 × 10^2^	1.4 × 10^3^	6.2 × 10^5^	4.0 × 10^2^	5.0 × 10^2^
FC	4.4 × 10^4^	2.6 × 10	2.2 × 10^2^	3.4 × 10^4^	—	—
*F. streptococcus*	2.0 × 10^4^	2.1 × 10	2.1 × 10^2^	6.0 × 10^3^	—	—
*E. coli*	2.0 × 10^3^	1.2 × 10	4.4 × 10	6.6 × 10^2^	—	—

**Table 3:** Bacteriological characteristics of Karu abattoir wastewater, source of water used at the abattoir, receiving water bodies upstream and downstream, TC effluents limit for discharge into water bodies and land application in Nigeria (MPN/100 mL).

Parameter	Mean value of abattoir wastewater	Mean value of water used at the abattoir	Mean value of water body upstream	Mean value of water body downstream	^ ∗^Effluent limit for discharge into surface water in Nigeria	^ ∗^Effluent limit for discharge into land application in Nigeria
TC	4.8 × 10^6^	2.0 × 10^3^	5.2 × 10^3^	6.6 × 10^5^	4.0 × 10^2^	5.0 × 10^2^
FC	8.2 × 10^4^	1.6 × 10^2^	3.4 × 10^2^	6.2 × 10^4^	—	—
*F. streptococcus*	5.2 × 10^4^	1.0 × 10^2^	1.2 × 10^2^	1.8 × 10^4^	—	—
*E. coli*	1.2 × 10^4^	2.8 × 10	2.0 × 10^2^	4.8 × 10^3^	—	—

**Table 4:** Bacteriological characteristics of Kubwa abattoir wastewater, source of water used at the abattoir, receiving water bodies upstream and downstream, TC effluents limit for discharge into water bodies and land application in Nigeria (MPN/100 mL).

Parameter	Mean value of abattoir wastewater	Mean value of water used at the abattoir	Mean value of water body upstream	Mean value of water body downstream	^ ∗^Effluent limit for discharge into surface water in Nigeria	^ ∗^Effluent limit for discharge into land application in Nigeria
TC	3.2 × 10^6^	2.0 × 10^2^	2.6 × 10^3^	6.1 × 10^5^	4.0 × 10^2^	5.0 × 10^2^
FC	3.2 × 10^4^	1.0 × 10^2^	3.8 × 10^2^	1.8 × 10^4^	—	—
*F. streptococcus*	3.1 × 10^4^	2.4 × 10	2.0 × 10^2^	1.4 × 10^4^	—	—
*E. coli*	5.4 × 10^3^	2.0 × 10	1.0 × 10^2^	3.0 × 10^3^	—	—

**Table 5:** Bacteriological characteristics of Kuje abattoir wastewater, source of water used at the abattoir, receiving water bodies upstream and downstream, TC effluents limit for discharge into water bodies and land application in Nigeria (MPN/100 mL).

Parameter	Mean value of abattoir wastewater	Mean value of water used at the abattoir	Mean value of water body upstream	Mean value of water body downstream	^ ∗^Effluent limit for discharge into surface water in Nigeria	^ ∗^Effluent limit for discharge into land application in Nigeria
TC	4.4 × 10^6^	4.8 × 10^3^	5.1 × 10^3^	6.0 × 10^5^	4.0 × 10^2^	5.0 × 10^2^
FC	4.2 × 10^4^	4.0 × 10^2^	2.4 × 10^3^	2.8 × 10^4^	—	—
*F. streptococcus*	2.8 × 10^4^	1.8 × 10^2^	1.2 × 10^3^	1.2 × 10^4^	—	—
*E. coli*	2.4 × 10^3^	4.0 × 10	1.0 × 10^2^	1.8 × 10^3^	—	—
